# Gender, sexuality and the discursive representation of access and equity in health services literature: implications for LGBT communities

**DOI:** 10.1186/1475-9276-10-40

**Published:** 2011-09-29

**Authors:** Andrea E Daley, Judith A MacDonnell

**Affiliations:** 1 School of Social Work, Faculty of Liberal Arts and Professional Studies, York University, Toronto, ON, M3J 1P3, Canada; 2 School of Nursing, Faculty of Health, York University, Toronto, ON, M3J 1P3, Canada

## Abstract

**Background:**

This article considers how health services access and equity documents represent the problem of access to health services and what the effects of that representation might be for lesbian, gay, bisexual and transgender (LGBT) communities. We conducted a critical discourse analysis on selected access and equity documents using a gender-based diversity framework as determined by two objectives: 1) to identify dominant and counter discourses in health services access and equity literature; and 2) to develop understanding of how particular discourses impact the inclusion, or not, of LGBT communities in health services access and equity frameworks.The analysis was conducted in response to public health and clinical research that has documented barriers to health services access for LGBT communities including institutionalized heterosexism, biphobia, and transphobia, invisibility and lack of health provider knowledge and comfort. The analysis was also conducted as the first step of exploring LGBT access issues in home care services for LGBT populations in Ontario, Canada.

**Methods:**

A critical discourse analysis of selected health services access and equity documents, using a gender-based diversity framework, was conducted to offer insight into dominant and counter discourses underlying health services access and equity initiatives.

**Results:**

A continuum of five discourses that characterize the health services access and equity literature were identified including two dominant discourses: 1) multicultural discourse, and 2) diversity discourse; and three counter discourses: 3) social determinants of health (SDOH) discourse; 4) anti-oppression (AOP) discourse; and 5) citizen/social rights discourse.

**Conclusions:**

The analysis offers a continuum of dominant and counter discourses on health services access and equity as determined from a gender-based diversity perspective. The continuum of discourses offers a framework to identify and redress organizational assumptions about, and ideological commitments to, sexual and gender diversity and health services access and equity. Thus, the continuum of discourses may serve as an important element of a health care organization's access and equity framework for the evaluation of access to good quality care for diverse LGBT populations. More specfically, the analysis offers four important points of consideration in relation to the development of a health services access and equity framework.

## Background

Public health and clinical researchers across a number of countries agree that there is now strong evidence to show that as a population, LGBT people (The acronym LGBT is used in some areas of the paper as a means of including the broad spectrum of sexual and gender identities and communities. During other areas of the paper other acronyms are used, e.g., LG, when referencing research that focuses on the health care experiences of members of some sexual and gender identities and communities and not others) experience significant health inequities with well-documented negative health impacts that include increased risks for chronic disease and mental health concerns [1-8]. Subgroups of LGBT people across age, ethnicity, and other social locations, (e.g, bisexuals, Aboriginals) also encounter unique issues which are linked to a variety of social determinants of health including the intersection between gender, race, sexuality, socioeconomic status and employment [9-12]. Research suggests that despite the criterion of universality and accessibility that anchor health care delivery in Canada, LGBT people do not always receive health services on uniform terms and conditions, and that the provision of health services may impede or preclude their reasonable and equitable access.

Over the last 15 years, a growing body of Canadian research has focused on barriers to equitable access to health and social services for LGBT people. This literature represents an articulation of LGBT communities' health issues that has emerged from several distinct, but interrelated sources. Community activism within and across LGBT communities produced seminal documents related to access to care [13-16]. Academic researchers in health and social sciences have explored access for a range of population groups including seniors, Two-Spirited people, and lesbian/queer women [9,17-22]. More recently, professional bodies such as the Canadian Professional Association for Transgender Health (CPATH) and the Registered Nurses Association of Ontario [RNAO] have taken leadership roles to raise awareness about LGBT access to care.

Everyday threats to LGBT people's health have been linked to heterosexism, biphobia, and transphobia which are embedded in all social instutions and which contribute to social exclusion, stigma and discriminatory dynamics, as well as invisibility and lack of health provider knowledge and comfort [13,23]. LGBT people of diverse ages, ethnicities, religions and geography represent up to 5-10% of the population. In the province of Ontario (Canada), there are thus an estimated 400, 000 to 1.25 million LGBT people who may anticipate and face barriers to access to health programs and services [23-25].

With an increasing awareness of the need to address the health concerns of diverse populations, including LGBTs, health policy bodies have called for the reporting of equity initiatives undertaken by health care institutions. In Ontario, Canada, for example, this past year, hospitals in the Toronto Central Local Health Integrated Network (TCLHIN), a local health authority responsible for planning, coordinating and funding key health care services for approximately 1.14 million people in central Toronto, were required to report on equity initatives [26,27]. Similarly, public health committees and health equity councils have produced documents that explore the delivery of quality health care to diverse populations experiencing health disparities.

Importantly, while processes that foster access and equity for LGBT people are moving forward in hospital, long-term care and public health sectors, similar initiatives have overlooked the home care sector. In response to this identified gap, our initial intent was to explore the extent to which the home care sector access and equity literature addresses access and equity in the provision of in-home health services for members of LGBT communities. However, a literature search failed to yield health services access and equity frameworks developed specifically for the home care sector and the provision of in-home health services in relation to diversity generally, and LGBT populations specifically. One document provided a comprehensive toolkit for enhancing equitable access and good quality care for LGBT older people within institutional residential settings (e.g., long term care facilities) [28]. The toolkit identifies important areas of consideration for providing care to LGBT people; however, it may be more aligned with institutional (hospital-based) strategies and frameworks, while failing to consider the unique access issues for organizations providing care to LGBT people within private home settings. In the absence of home care-specific access and equity literature, our focus shifted to providing a critical discourse analysis (CDA) of selected health services sector access and equity documents generally, using a gender-based diversity framework as determined by two objectives: 1) To identify dominant and counter discourses in health services access and equity literature; and 2) to develop understanding of how particular discourses impact the inclusion, or not, of LGBT communities in health services access and equity frameworks.

## Methods

A critical discourse analysis of selected health services sector access and equity documents, using a gender-based diversity framework, was conducted to offer insight into dominant and counter discourses underlying organizational access and equity initiatives. As a method of social science research, critical discourse analysis is useful for probing underlying philosophical assumptions, ideological commitments and implicit knowledge-power dynamics underlying organizational texts [29]. CDA centres the role of language in organizational texts to "establish identities, social relationships and systems of knowledge and belief" [29]. Critical discourse analysis can reveal structures of domination and control including, for example, how dominant groups in contemporary organizations may inadvertently control diversity issues in a way that privileges some groups while marginalizing others [30]. In this regard, social practices within organizational settings are discourse-led practices that "can set the parameters and the conditions of possibility, for what can be perceived, articulated, and experienced" [31]. This has important implications for which access and equity issues are identified as relevant to care and the strategies that are created in response to them.

The term "gender-based diversity analysis" highlights the importance of examining intersections among racialization and other social processes such as sexuality and gender identity which are simultaneously implicated in the way relations of care are structured and experienced [32-34]. It offers insight into the contradictory and complex dynamics which shape the lives of differently situated women and men, including transgender women and men [35,36]. Gender and diversity are thus linked to access to meaningful and responsive programs and services [37] and offer a deeper insight into discourses that operate in the textual documents.

### Search Strategy

A keyword search was conducted of medical, psychology, health sciences, social science, and sociological electronic databases to locate peer reviewed literature on health services access and equity. A 'funnel-approach' to the search was taken that began with the use of single and combined broad-based terms such diversity, cultural competency, access, equity, equality, health services, social services, health policy, social policy and public policy. Next, in an effort to narrow the search LGBT- specific single and combined terms were used including sexual diversity, sexual orientation, sexual minority, sexual identity, gender identity, and homosexual. In addition to the search of electronic databases, Canadian, US and international health-oriented internet sources were searched to identify relevant health services sector access and equity grey literature. The search included health care organizations' and LGBT health and social services' web sites; heath research institutes, health service provider associations, colleges and unions and/or affiliated LGBT working groups or caucuses; and LGBT health and social services' grassroots activist groups, coalitions and LGBT-specific health and social care professional associations. In an effort to capture the impact of recent legislative changes (e.g., same sex marriage and employment legislation in Canada, UK, Europe and select US states) on health services policy and practices, the search covered 1995-2009.

The literature search yielded: 1) Peer-reviewed empirical research and theoretical papers from medical, psychology, health sciences, social science, and sociological academic journals that reported on health disparities, health services access barriers and experiences, and experiences associate with self-disclosure during care interactions for LGBT people; 2) health service organization (e.g., hospitals, public health) health equity reports and access and equity frameworks and measures related to diversity; 3) health research institute discussion papers exploring health equity and diversity; and 4) LGBT group, association and network-developed LGBTQ-specific health service access and equity frameworks.

The health services access and equity literature related to diversity included in this analysis is largely limited to Ontario- and Toronto-based health equity position papers, reports and frameworks that articulate a vision of enhancing equitable access to health services and how to achieve it in practice. The decision to limit inclusion to Ontario- and Toronto-based health equity documents was based on their proliferation as a result of an increased awareness, over the past several years, of health disparities and the need to account for diversity in health policy and care delivery in Ontario [26,27].

The health services access and equity literature related to sexual and gender diversity included in the analysis is broader in geographical scope than the health services access and equity literature related to diversity in general. The decision to expand the inclusion criteria to include Canada-wide, US and international literature is largely based on the limited availability of 'home grown' LGBT-specific health services access and equity literature. Canada generally, and Ontario specifically, has offered significant contributions to dialogues towards the advancement of equitable access and good quality care for LGBT communities. For example, policy bodies and professional associations such as the Ontario Public Health Association [14-16], RNAO [25] and CPATH have called for system-level reform to improve LGBT access to care. Additionally, in 2008, the Ontario Ministry of Health and Long-Term Care funded *Rainbow Health Ontario*, whose mandate is to improve the health and well-being of lesbian, gay, bisexual and transgender people in Ontario through education, research, outreach and public policy advocacy [38]. However, we include significant international work related to LGBT health services access and equity where relevant.

Selected documents were read and re-read by two research team members (AD, JM) using the following elements to guide the analysis: 1) representations of health, access, culture and diversity; 2) representations of gender, sexuality, race, class and ability; and, 3) absences or silences related to gender, sexuality, race, class and ability. In terms of 'representations' documents were reviewed for the ways in which particular language and/or terms were used to identify population-based health disparities and barriers to health services; determine and demarcate populations and communities based on social identities and locations; and articulate potential solutions towards increased access to good quality health care. 'Absences or silences' refers to the identification of the omission of language and/or terms for some health disparities and access barriers to health services for some populations and communities. Based on this process, organizational assumptions, knowledge and commitments underlying access and equity documents, as reflecting multiple and competing dominant and counter discourses within and across texts were identified.

## Results

A total of twenty-four (24) health services access and equity documents that address the provision of institutionally-based health care were selected for review and analysis. Selected documents were categorized as addressing access and equity related to diversity generally (n = 10) and access and equity addressing sexual and gender diversity (LGBT) specifically (n = 14). The documents derive from Canada, the US and Scotland and are representative of government and local health authorities (6), government in collaboration with LGBT- specific associations (3) hospitals (3), public health departments and associated committees (5), non-LGBT- and LGBT-specific health-related professional associations and unions (3) and LGBT-specific services (2). Two (2) documents constitute government documents that focus on LGBT inclusion in multi-sector policy and planning broadly while serving as integral guiding documents for health services access policy and planning. While these documents may not appear to adhere with the search strategy focus on the health services sector access and equity literature adopted for this analysis, their inclusion is nevertheless important given their multi-sectoral purpose and potential to influence and guide regional and organizational health services access and equity frameworks.

**Figure 1:**
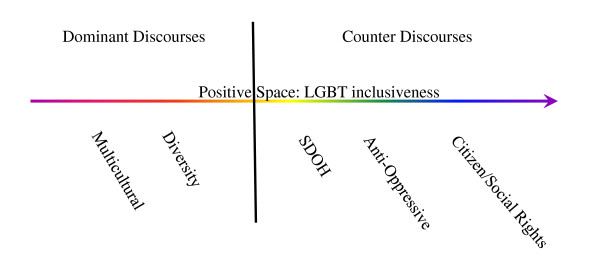
**Dominant and Counter Discourses**.

A continuum of five discourses that characterize the health services access and equity literature were identified including two dominant discourses: 1) multicultural discourse, and 2) diversity discourse; and three counter discourses: 3) social determinants of health (SDOH) discourse; 4) anti-oppression (AOP) discourse; and 5) citizen/social rights discourse (Figure 1). Importantly, the term 'continuum' reflects the fluidity, blending and complexity of language use in health services access and equity texts as it relates to any one identified discourse within the context of multiple audiences (e.g., health care service providers, service users/consumer groups, health authorities and policy decision-makers), organizational and political contexts (religious) and expectations (e.g., reducing health disparities, enhancing social equity). In addition, the term 'continuum' suggests that while the exploration and analysis of the identified discourses may construct each as both separate and operating separately from each other, we conceptualize dominant and counter discourses as existing along a continuum of variation in degree and often operating simultaneously within and across the selected access and equity literature. This dynamic is captured through the groupings of multicultural and diversity discourses and social determinants of health and anti-oppression discourses in the analysis presented below. Each discourse will be explored in turn by juxtaposing dominant and counter discourses operating in the health services access and equity literature related to diversity generally, and sexual and gender diversity specifically.

### Multicultural and Diversity Discourses

#### Cultural competence

While the health services access and equity literature that aims to address diversity contributes important elements to enhancing equitable and good quality care for diverse and vulnerable populations, a major limitation is the non-representation or marginal representation of LGBT populations. A majority of the documents focus predominantly on the important but limited issue of health services inequity among different language and racial/ethnic groups. While a focus on language and racial/ethnic groups does not immediately and necessarily exclude LGBT people, it is often indicative of a dominant multicultural discourse that operates within the health services access and equity literature addressing diversity. For example, one document that fails to explicitly define culture in relation to service access implicitly delimits the term to race and language vis-à-vis cited examples of health inequities that do not include references to LGBT communities. In this regard, health services access barriers and inequity is typically framed as a cultural incompetence issue, on the part of health care service providers, to be redressed through acknowledgement that patients and hospital staff can bring unique points of view to health care interactions, based on their experiences, culture, and communities. Strategies to enhance culturally competent practices, or rather culturally congruent care, among health care service providers is typically implied in regards to multicultural communities vis-à-vis the development of cultural competency key indicators, for example, redressing communication barriers related to language through the development of language services. Importantly, the very few references to LGBT populations are often bracketed as afterthoughts or a consideration for future organizational initiatives.

#### Broadening the culture discourse

Also operating within the health services and access literature on diversity, either independent of or in conjunction with the multicultural discourse, is a dominant diversity discourse. The diversity discourse represents a broader conceptualization of culture in access and equity frameworks as demonstrated, for example, by organizational statements that recognize *any *group that has a culture of its own. Health services access and equity documents that adopt a diversity discourse are more likely to recognize LGBT communities as cultural communities by, for example, including statements related to existing evidence that LGBT-identified people face barriers to accessing health care services; identifying LGBT groups as a vulnerable population; and, addressing LGBT people in organizational equity statements. For example, while documents may addresses equitable health services by emphasizing "continuing efforts to reduce disparities in the health of those groups who may be disadvantaged by social or economic status, age, gender, ethnicity, geography, or language" [39], others recognize that although this is a "good starting point for shaping health care policy and delivery, it can be broadened further to include health disparities related to racism and discrimination, culture, citizenship status, *sexual orientation*, and ability" (our emphasis) [40]. Of additional significance to this excerpt is the use of the language 'racism and discrimination' as a means of suggesting a shift along the continuum towards both the social determinants of health (SDOH) and anti-oppression counter discourses, which typically centres the individual social identities of service users and service providers and systemic oppressions in relation to health services access barriers. This excerpt also demonstrates how a dominant diversity discourse may only minimally represent LGBT people by offering a fleeting reference to these populations vis-à-vis the inclusion of *sexual orientation *as a category of diversity.

Within the health services access and equity literature related to sexual and gender diversity the diversity discourse also operates; however, it does so largely in the absence of the dominant multicultural discourse. Within these texts, the diversity discourse operates through the textual representation of LGBT communities as a distinct, cultural minority community embedded within the dominant heterosexual culture and a stated need for service providers to understand (and embrace) standards of practice for the provision of quality health care services to LGBT communities. Following this, documents often redress access barriers to health services for LGBT people through the training of service providers towards LGBT diversity competent care by, for example, using inclusive language (e.g., partner versus husband or wife) and avoiding assumptions about gender and sexual identity.

Importantly, some documents suggest that service providers may demonstrate LGBT diversity competent care towards the avoidance of assumptions about sexual identity by asking questions about sexual behaviour rather than sexual identity. Conceivably, this strategy might be expected and encouraged within the context of a Western biomedical model of health that separates the body from the mind and that privileges the material (body) over the social (identity). The dominance of a Western biomedical model of health in current debates related to health services access is well documented. For example, recently developed benchmarks for measuring access to care, such as, wait times for joint replacement, the percent of the population with a regular family physician and access to medical advice afterhours, have been critiqued for their narrow biomedical perspective on health disparties which overlooks the complex structural dynamics and social determinants that influence equitable access to care for diverse groups, especially those who are on the margins of society [32,35,37]. These types of access and equity indicators appear to be developed in relation to identified priority populations based on limited demographic characteristics, disorders and diagnoses meaning that 'access' is often textually represented in concrete ways related to health services resource allocation.

While LGBT diversity competency strategies such as the one described above, that emerge from the biomedical model, may well be successful in terms of avoiding assuming questions, they often negate the significance of identity - recognition, acceptance and affirmation - to overall health and well-being for LGBT people [41]. In addition, it fails to recognize LGBT people who attempt to access the health care system in response to psycho-social distress (e.g., mental health services) rather than physical disease (not that there is always a clear distinction between the two), which may be related to, and therefore centre, issues of identity, relationships and community with less attention to sexual and reproductive health and behaviour. Moreover, this strategy risks individualizing population health issues while failing to capture the interaction between the material (body) and social (identity) for LGBT people who have historically, and continue to, experience the regulating of their (queer) bodies and minds. This may be particularly relevant to transgender people within the context of particular mental health and psychiatric settings.

Of equal importance, the operation of a diversity discourse in the LGBT health services access and equity literature often fails to address diversity in LGBT communities in relation to racialized, classed, and differently-able bodies and minds. In this regard, these documents also function to homogenize LGBT communities while risking normative representations (e.g., white, middle-class) of racialized, classed and able-bodied and minded LGBT people.

### Social Determinants of Health (SDOH) and Anti-oppression (AOP) Discourses

While much of the health services access and equity literature on diversity reflects the Western biomedical model of health as described above, some public health documents prioritize the social determinants of health (SDOH) and anti-oppression discourses as counter discourses to the dominant multicultural and diversity discourses. The operation of the SDOH and anti-oppression discourses within health services access and equity texts is typically represented within documents that offer of a broad vision of organizational change towards enhancing access to good quality care beyond that of making diversity competent practitioners. As such, structural power and the need to shift power through structural transformation is recognized as integral to enhancing health services access for diverse vulnerable populations. For example, some documents clearly identify that some people do not get care because of who they are while underscoring the imperative to expand access initiatives to respond to health disparities related to discrimination, such as racism. One organizational document states, for example, "providing effective and efficient services to the diverse population of Ontario requires more than diversity competency training for public health practitioners. The development of inclusive policies and procedures, and the hiring of workers from diverse groups are examples of other strategies needed in building a diversity competent organization" [42].

#### Intersectionalities

The operation of the SDOH and anti-oppression discourses within access and equity documents related to diversity has the potential to position sexual and gender identity, and their associated structural inequities including systemic oppressions and social stigma as implicated in the health and wellbeing of LGBT people and communities. However, as with the dominant diversity discourse explicit examples of structural change related to sexual and gender diversity is largely absent from health services access and equity documents that address diversity generally. For example, one document identifies accessibility, as an equity key indicator, as receiving appropriate care in the most appropriate setting while acknowledging that access to a range of health services continues to be a problem for some people including the poor, immigrants and rural residents to the exclusion of LGBT communities. This limitation is more likely to be redressed by the few health services access and equity documents related to diversity that are more strongly aligned with an anti-oppression discourse through the identification of access initiatives that textually represent health disparities as a result of intersecting forces (e.g., race, immigration status, education).

Within these documents there is increased recognition of the vulnerability of diverse LGBT populations. However, while the uptake of intersectionality in documents as represented by the anti-oppression discourse improves the possibility of having diverse LGBT populations represented as vulnerable populations, they may only minimally include examples of measures that specifically address LGBT vulnerability. For example, one document that makes reference to LGBT vulnerability and that provides a list of definitions related to potential health disparities for diverse populations including culture, diversity, bias, oppression, racism fails to include language and definitions that speaks directly to health disparities and health services access barriers for LGBT communities, such as, homo/bi/lesbi/transphobia, heterosexism, and genderism. In addition, many LGBT-specific statements accompanying access and equity frameworks refer to LGBT people as a homogenous group in the absence of recognition of the differential experiences with health services access related to intersecting identities based on gender and race, among others.

#### Material and social implications: Cautions about reductionism

Arguably, the SDOH and anti-oppression discourses require that health care providers develop understanding that 'who' people are (social identity) may be related to their experiences of physical health, thereby, recognizing the intersection between the social (identity) and material (physical). This would mean, for example, that health services access and equity documents related to income and health inequities that propose health-related behaviour (smoking, physical inactivity) measures as key indicators consider, for example, inceased smoking rates among lesbian women in relation to minority stress [43,44]. Doing so, would acknowledge that smoking is but one way of coping with the tensions of living in a heteropatriarchal society and the associated violences of lesbophobia and homophobia. As such, the SDOH and anti-oppression discourses may avoid the individualism of a diversity discourse that runs the risk of reducing population health issues to individual lifestyle/behaviour.

Within the health services and access literature related to sexual and gender diversity, the SDOH and anti-oppression discourses often operate through 'positive space' strategies within organizations that expand change initiatives to include broader organizational practices and structures in an effort to enhance access to good quality care for LGBT populations. As a counter discourse to the dominant heteronormative mulitcultural discourse, and to some extent the diversity discourse, the anti-oppression discourse operating within these texts extends the requirements for equitable access and good quality care for LGBT communities to include information and key indicators that address structural issues. This includes, for example, issues of governance, administration, personnel policies and practices, communication, community relations and health promotion, service delivery, physical environment and organizational culture. Of critical importance then, LGBT people and communities are not only represented as users of health care services but also as health services administrators and other health care personnel, health care service providers and engaged citizens.

### Citizen/Social Rights Discourse

Other LGBT-specific health services access and equity documents offer a counter discourse to the dominant mulitcultural and diversity discourses, and to some extent the SDOH and AOP discourses by adopting a citizen/social rights discourse. For example, the National Health Services in the UK developed a comprehensive guidance document that gives practical advice to NHS organizations to help them implement and comply with the requirements of legislation on sexual orientation enacted, which gives rights to equal treatment regardless of sexual orientation [45]. The UK Equality Act (Sexual Orientation Regulations) 2007 makes it unlawful to discriminate on the grounds of sexual orientation in the provision of goods, facilities and services and the exercise of public functions in both the private and public sectors, including healthcare [45]. The equity recommendations and strategies developed for the health sector are part of a larger multi-sectoral focus on enhancing social right to service access for LGBT citizens that includes the education and employment sectors. Similarly, a document published by the Scottish Executive assists Councils to assess progress in developing policies and practice in relation to LGBT people and practical suggestions about good practice [46]. This document provides ideas about how councils can be more responsive to LGBT communities (Part 1) and a checklist which can be used to assess how a council has developed its approach to LGBT people (Part 2) [46].

Within the Canadian context Quebec's Anti-Homophobia Policy [47] may function similarly to the UK Equality Act in underscoring the "rights of sexual minority people to participate fully in all aspects of life in society", the "state's role as a leader in upholding rights and freedoms" for LGBT citizens and the "responsibility and commitment of all institutions" to combat homophobia [47]. The policy is structured by four guidelines each with respective strategic choices. Of interest is Guideline 2, "promoting respect for the rights of sexual minority members" and Guideline 4, "ensuring a concerted approach" [47]. The first and second strategic choices of Guideline 2 include promoting sexual minority member rights through strong recognition of the rights of sexual minority members and helping LGBT people exercise their rights through the creation of resources to help those who experience homophobia defend their rights. The identified strategic choices for Guideline 4 include ensuring synergy between initiatives of the government and other public institutions and the support of local support and regional authorities and other government partners to fight homophobia.

Currently, the Anti-Homophobia Policy is beginning to be operationalized through the development of an interdepartmental committee with minister-appointed delegates from all areas of government including public security, health and social services, education, sports and leisure, family and the elderly, culture, communication and condition of women, immigration and cultural communities, labour, employment and welfare [47]. As such, the policy holds the potential to serve as a guidance document for health care institutions, in the province of Quebec, towards enhanced health services access and good quality care for LGBT citizens. Caution must be taken, however, in that the policy document appears to conflate sexual orientation and gender identity through the textual representation of transsexual and transgender people as sexual minorities. This is an important oversight that can have critical consequences related to the ability of the document to provide guidance towards equitable transgender health services access. Moreover, the conflation of sexual orientation and gender identity risks the perpetuation of ongoing trans exclusion in considerations of health services barriers and access, and associated initiatives that may inadvertently address LGB populations only.

## Discussion

This paper has applied a critical discourse analysis using a gender-based diversity framework to health services access and equity documents that address both diversity generally, and sexual and gender (LGBT communities) diversity specifically. We recognize that documents have been created often with a strategic purpose in mind. Thus, the aim is not to critique particular documents per se, but to examine and understand the multiplicity and competing discourses in such documents. The objectives of the analysis were to develop understanding of dominant and counter discourses operating in the health services access and equity literature, and the implications of the discourses for LGBT health services inclusion/exclusion. To this end two dominant discourses: 1) Multicultural discourse; and 2) diversity discourse; and three counter discourses: 3) social determinants of health (SDOH) discourse; and 4) anti-oppression (AOP) discourse; and 5) citizen/social rights discourse are identified in relation to the textual representations, or not, of LGBT communities. The analysis offers four important points of consideration in relation to the development of health services access and equity frameworks.

First, and most notable, the continuum of discourses provides a lens through which to assess whether and to what extent LGBT people and health are considered and included in organizational health access and equity initiatives. This would include using the continuum of discourses (see Figure 1) to interrogate underlying organizational assumptions and ideological commitments as reflected in the language of organizational access and equity frameworks (multicultural, diversity, social determinants of health, anti-oppression, and citizen/social rights discourses). This approach prompts questions that can move organizations to consider strategies for change. Which communities or populations are implied in the access and equity framework, and which communities or populations are not implied? What organizational and contextual forces contribute to the development of the framework, and inclusion or exclusion of particular communities or populations? And, how has the organization's use of the framework in particular ways shaped which access and equity initiatives have been identified as relevant, inclusive and effective? The identification of organizational access and equity discourses draws attention to social practices within organizations, and the implications of their practices for LGBT communities and health services access.

Second, and related to the first point of consideration, the continuum of discourses suggest that within an organizational context multiple and competing discourses may operate. Of particular significance, however, is the use of multiple and competing discourses *with intention *as a strategic approach and response to contextual tensions. Factors such as the religious associations of some health care organizations that do not condone 'homosexual' behaviour, funding requriements, local health authority policy and planning in relation to identified priority populations, and external advocacy pressures can all play a part in shaping how organizations position themselves strategically in particular times and places as reflected through such documents.

For example, it is conceivable that the coupling of a multicultural discourse that offers little, if any, representation of LGBT health and populations and a diversity discourse that offers increased representation of a homogenized LGBT population with no representation of its diversity may reflect political tensions as they exist within organizations. This may include, for example, tensions resulting within urban health service organizations that must adhere to religious and political doctrines that fail to create the space for representations of LGBT communities while providing services to LGBT communities. It may be related to the decisions and funding requirements of local health policy and planning authorities that prioritizes some marginalized populations to the exclusion of others. Tensions may also be reflective of change processes resulting from the advocacy work of LGBT employees and allies within health care organizations, LGBT health activists and other forms of external pressure from LGBT communities. In short, the operation of these competing discourses may more accurately be understood as an organizational strategy towards the incremental creation of space for LGBT communities within the context of religious and/or political and policy tensions.

The continnum of discourses may serve as a navigational tool whereby organizations may identify short-term, mid-term and long-term access and equity objectives and their associated strengths and limitations towards the development of initiatives that encourage the recognition, acceptance and affirmation of LGBT people through the creation of positive space (anti-oppression discourse) and that satisfy the social right to health services access and good quality care for LGBT citizens (citizen/social rights discourse).

Third, the continuum of discourses may also serve as a lens through which organizations seek to recognize and address the issue of intersectionality in health services access and equity planning. That is, assessing how organizational assumptions underlying the use of terms such as culture, diversity and sexual and minorities capture, or fail to capture, overlapping oppression including, for example, LGBT people within classed and racialized categories and classed and racialized people within LGBT categories. A consideration of the continuum of discourses that characterize the health services access and equity literature requires that access and equity frameworks respond to the layered ways in which multiple social identities and systemic oppressions and associated health services access barriers exists within vulnerable communities, such as LGBT communities (anti-oppression discourse).

Fourth, the continuum of discourses provides an important framework for exploring organizational understandings of LGBT identities in relation to LGBT health and well-being and health services access. As the analysis suggests, a diversity discourse with its primary focus on creating diversity competent service providers may ignore population health issues for LGBT communities. An exclusive focus on an LGBT individual's behaviour without attending to identity-based experiences of social discrimination and exclusion may mean that organizations fail to recognize how their policies and practices may inadvertantly reproduce systemic heterosexism and genderism, and hence, health services access barriers for LGBT communities. The continuum of discourses may support health service organizations to recognize patterns within and across LGBT-identity categories related to health disparities and health services access. For example, the SDOH discourse that recognizes the impact of heterosexism and genderism as well as homo/lesbo/bi/transphobia would support a shift from asking about an individual's sexual behaviour to asking questions about sexual identity in relation to relationships, community and support. Moreover, an exclusive focus on individual behaviour (diversity discoures) is limited in terms of addressing health disparities for LGBT communities that are rooted in experiences of discrimination (SDOH discourse).

## Conclusion

This review and analysis of health services access and equity literature contributes to a growing body of research literature on the provision of accessible and equitable health services within a Canadian health service delivery context that is characterized by expanding community-based care. Applying a gender-based diversity perspective to this analysis is an important first step towards enhancing ethical care and equity health services for LGBT communities. In this way, this approach is consistent with Health Canada and the WHO's calls for integrating a gender-based analysis into health and policy research [48,49] with a goal of strengthening the scientific knowledge base which will "enhance health outcomes and strengthen health care" [34] for LGBT communities.

## Competing interests

The authors declare that they have no competing interests.

## Authors' contributions

AD and JM engaged in a collective process of reviewing and selecting health services access and equity documents included in the analysis. AD and JM engaged in a collective process of identifying dominant and counter discourses operating in the selected documents. AD drafted the manuscript with input and revisions from JM. AD provided interim and final formatting. AD provided a first draft of revisions based on reviewer comments. JM provided review of revisions and amendments based on reviewer comments. Both authors read and approved the final manuscript.
